# Determining the Importance of Carbohydrate-Based Structures in Murine Norovirus Binding to Commensal Bacteria

**DOI:** 10.3390/v17081142

**Published:** 2025-08-20

**Authors:** Jasmine L. Madrigal, Joseph P. Sullivan, Feba Mathew, Melanie Bland, Melissa K. Jones

**Affiliations:** Department of Microbiology and Cell Science, IFAS, University of Florida, Gainesville, FL 32611, USA; jlmadrigal1@yahoo.com (J.L.M.); josephsullivan1@ufl.edu (J.P.S.); feba.math@gmail.com (F.M.); lanebarnes17@gmail.com (M.B.)

**Keywords:** human norovirus, murine norovirus, carbohydrate-based interactions, histo-blood group antigens, glycans, HBGAs, aminoglycosides, GlcNaC, viral attachment, virus–bacterial interactions, MNV P2 domain, fucose, flagella

## Abstract

Norovirus–bacterial interactions influence viral replication and immune responses, yet the molecular details that mediate binding of these viruses to commensal bacteria are unknown. Studies with other enteric viruses have revealed that LPS and other lipid/carbohydrate structures facilitate virus–bacterial interactions, and it has also been shown that human noroviruses (HuNoVs) can interact with histo-blood group antigen (HBGA)-like compounds on the surface of bacterial cells. Based on these findings, this study hypothesized that carbohydrate-based compounds were the ligands that facilitated binding of both human and murine noroviruses (MNV) to bacteria. Using glycan microarrays, competitive inhibition assays, and a panel of bacterial mutants, the project assessed the influence of specific glycans on viral attachment to bacteria. Protein-based interactions were also examined. The results supported previous work which demonstrated that HuNoVs strongly bind HBGA-like glycans, while MNV displayed distinct binding to other glycans including aminoglycosides and fucosylated structures. Ultimately, this work demonstrates that HuNoVs have more limited binding requirements for bacterial attachment compared to MNV, and the MNV binding to bacteria may involve both specific structures as well as electrostatic interactions. Given the importance of commensal bacteria during viral infection, defining the molecular mechanisms that mediate virus–bacteria interactions is critical for understanding infection dynamics and may be useful in the development of disease therapeutics and novel technologies for viral detection from food and environmental sources.

## 1. Introduction

Human noroviruses (HuNoVs) are the leading cause of gastrointestinal disease worldwide, causing approximately 1 billion infections annually and associated costs exceeding $60 billion [1,2,3]. Despite their impact, HuNoVs remain challenging to study due to limited cultivation systems [4,5,6,7], which necessitates the use of surrogate viruses such as murine norovirus (MNV). A growing body of evidence underscores the critical interplay between enteric viruses and commensal bacteria, including noroviruses, rotavirus, coxsackie virus, and poliovirus [4,8,9,10]. These bacteria modulate enteric viral infections through a variety of mechanisms such as enhancing viral binding, promoting viral stability, influencing immune responses, and improving viral transmission [8,10,11,12,13,14]. Commensal bacteria offer a rich environment for viral particles to bind, protecting the virus from environmental degradation and host defenses [15,16,17,18]. This virus–bacterial interaction can also lead to modification of host immune responses that can aid or hinder viral replication depending on the virus and other factors [10,14,19,20]. The relationship between commensal bacteria and enteric viruses is complex, and the mechanisms through which bacteria modulate viral infection varies among viruses [8,10,11,12,13,14].

Noroviruses were among the first found to bind directly to commensal bacteria with binding associated with the presence of histo-blood group antigen (HBGA)-like compounds expressed on the bacterial surface [4,15,21]. It is well-established that HBGAs are involved in HuNoV infection [22,23,24,25]; therefore, the presence of HBGA-like compounds on the surface of commensal bacteria suggests a role for these bacteria in viral infection. Like HuNoV, MNV binds to a wide variety of bacterial strains and both viruses also induce changes in bacterial gene expression and bacterial vesicle production [11,16,19]. Commensal bacteria influence MNV infection in adult mice in a region-specific manner where the bacteria enhance viral replication in the distal regions of the gut but suppress replication in the proximal small intestine, likely due to bile acid-mediated immune priming [14], while in infant mice, microbiota differences based on feeding modes induce differential immune responses that can be associated with disease development [26]. These complex, site-specific, and age-specific differences in the microbial influence on norovirus infection illustrate the importance of defining bacteria–virus interactions so the mechanisms through which bacteria shape viral infection patterns can be clearly determined.

Investigations into MNV–bacterial interactions have found that specific bacterial structures such as lipopolysaccharide (LPS) and lipoteichoic acid (LTA) enhance MNV stability, but do not improve MNV infectivity [11]. Unfortunately, while these bacterial structures influence viral stability, the structures that mediate MNV attachment to bacteria have not been determined. Therefore, since HuNoVs binding to commensal bacteria is facilitated by HBGAs, we hypothesized that MNV-bacteria attachment would also be mediated by carbohydrate-based compounds. To test this hypothesis, we screened a broad glycan array to determine targets that may be facilitating norovirus–bacterial interactions and identified both shared and unique binding targets between HuNoV and MNV. Using these results, we performed targeted competitive inhibition assays to determine the influence of identified carbohydrate compounds in norovirus attachment to commensal bacteria. Overall, results confirmed previous work showing that HuNoVs bind to HBGAs and revealed that HuNoVs interact with a large number of complex carbohydrate structures. Indeed, only compounds containing HBGA-like carbohydrates impacted HuNoV-bacterial binding while none of those same compounds altered MNV–bacterial binding, indicating different structural requirements for bacterial binding between the two viruses. We noted that many of the glycans bound by MNV possessed terminal glucose, fructose or galactose molecules. Thus, since complex carbohydrates did not influence MNV binding, we used competitive inhibition assays to examine the influence of terminal sugars on MNV–bacterial binding. The results showed that, like more complex carbohydrate structures, simple sugars also did not significantly reduce MNV–bacterial binding.

The lack of carbohydrate inhibition of MNV attachment to bacteria led us to investigate MNV attachment to protein rather than carbohydrate structures. Almand et al. [17,18], found that HuNoV VLPs bound to protein fractions of bacterial lysates containing outer membrane proteins such as OmpA. Therefore, using a panel of bacterial protein and carbohydrates deletion mutants, we measured MNV/bacterial attachment. Interestingly, MNV binding was not altered in any of the protein mutants but was significantly reduced in the *fucP* mutant, again pointing to carbohydrates mediating interactions between MNV and bacteria. Ultimately, these results demonstrate that noroviruses likely bind to carbohydrates on the surface of the bacterial cell with HuNoVs consistently binding to HBGA-like compounds while the specific structures bound by MNV are yet unidentified and may involve multivalent interactions that enhance virus binding affinity.

## 2. Materials and Methods

### 2.1. Bacterial Growth and Virus Production

*Escherichia coli* (ATCC 11229 and BW225113) was cultivated on Luria–Bertani (LB) agar plates at 37 °C. Single colonies were inoculated into LB broth and grown overnight (~18 h) with shaking at 200 rpm. Cultures were pelleted by centrifugation at 10,000× *g* for 7 min, washed twice with 1× phosphate-buffered saline (PBS). Bacterial concentration was estimated by taking the optical density at 600 nm and comparing to previously generated standard curves [5,15]. MNV-1 wild-type recombinant virus stocks were generated using pSPMNV-1.CW3 as previously described and stored at −80 °C [16]. Mutant MNV-virus stocks were kindly provided by Dr. Stephanie Karst [27,28]. HNoV virus-like particles (VLPs) were purchased from Creative Biosciences (Salt Lake City, UT, USA; #CBS-V700) and stored at −80 °C [16].

### 2.2. Glycan Microarray

Binding between carbohydrates and noroviruses was tested using murine norovirus (MNV) and HuNoV virus-like particles (VLP) to various glycan structures present in the Glycan Microarray 300 (Chemily, Peachtree Corners, GA, USA, Table S1). VLP and MNV samples were sent to Chemily and the manufacturer compared 30 μg each of MNV-1 and HuNoV GII.4 VLP (Creative Biosciences, Guangzhou, China) in parallel on the microarray in three trials. Experimental details provided by the manufacturer are as follows. Biotinylated IgG protein printed on the array was used as the positive control for these experiments. Spots containing only dilution buffer printed on the array acted as negative control. To prepare each sample, 36 μL of 1× labeling reagent was applied to samples at 1 mg total protein, then incubated with agitation at room temperature for 30 min. Three microliters of Stop Solution were then added to terminate the reaction. The microarrays were then dried, 400 μL of Sample Diluent, and incubated at room temperature for 30 min, and the microarrays were decanted. Next, the Biotin-labeled samples underwent centrifugation at 10,000 rpm for 5 min to remove impurities and then diluted. Biotin-labeled samples were added to each well with a volume of 400 μL each and allowed to incubate at room temperature for 3 h. Then, the plates were washed with 800 μL wash buffer for 5–7 min before adding 400 μL of dye-conjugated Streptavidin in the dark and incubating for 1 h at room temperature and washed 5× with 800 μL wash buffer with gentle agitation. Relative fluorescence intensity data was obtained via Axon GenePix laser scanner (Molecular Devices, San Jose, CA, USA). Each spot of glycan was measured for median background signal, subtracting local background values from the signal intensity values of each spot and then normalized to the first subarray using the equation.

### 2.3. Competitive Inhibition Assays

To perform competitive inhibition assays (CIA), concentrated stocks of carbohydrate targets in nuclease-free water were prepared using the following targets: LPS (Sigma Aldrich, Burlington, MA, USA; L3129), Peptidoglycan (Sigma Aldrich, Burlington, MA, USA; 69554), Mucin (Porcine Gastric Mucin, Sigma Aldrich, Burlington, MA, USA; M1778), N-acetyl-D-glucosamine (Sigma Aldrich, Burlington, MA, USA; A8625), N-acetylneuraminic acid (Sigma Aldrich, Burlington, MA, USA; A0812) at a concentration of 1000 ug/mL. Glucose, fructose, galactose and fucose solutions were also created using nuclease free water and were incubated with virus at the percentage concentrations indicated in the figures. All solutions were stored at −20 °C until use. Either ten micrograms of HuNoV VLP GII.4 or 1 × 10^7^ TCID_50_ of MNV-1 were mixed 1:1 (vol/vol) with a given carbohydrate stock solution and incubated for 1 hr at room temperature. For sialic acid and GlcNAc the molar ratio of compound to virus is 1.95 × 10^11^ and 2.72 × 10^11^, respectively. Experiments performed with fucose incubated MNV with 1 mg/mL or nuclease free water. *Escherichia coli* BW225113 stationary phase cultures were centrifuged at 10,000× *g* for 5 min and the pellet washed with 1× Phosphate-Buffered Saline (PBS). The wash step was performed twice followed by dilution to a concentration of 1 × 10^8^ CFU/mL. The virus-carbohydrate mixture was added to 1 mL of washed *E. coli*. These were incubated for 1 hr at 37 °C with gentle rotation. Following incubation, the bacteria were pelleted and washed 2× as described above. After a third centrifugation at 10,000× *g*, the bacterial pellet was analyzed for viral attachment. HuNoV VLP attachment was measured using flow cytometry. MNV attachment was measured using RT-qPCR.

### 2.4. Murine Norovirus–Bacterial Attachment Assay

To test viral binding to bacterial mutants, 1 × 10^7^ TCID_50_ of MNV diluted in dPBS was incubated with 1 mL of 1 × 10^8^ CFU of the *E. coli*. The wild-type and mutant strains used are listed in Table 1. Bacteria and virus were incubated for 1 h at 37 °C with gentle rotation. After incubation, the bacteria were centrifuged at 10,000× *g* for 5 min and washed twice with dPBS. MNV attachment was measured using RT-qPCR.

### 2.5. Flow Cytometry Quantification of HuNoV VLPs

HuNoV VLP attachment to bacteria was quantified using flow cytometry as we have performed before [5]. Norovirus capsid G2 monoclonal antibody (Thermo Fisher Scientific, Waltham, MA, USA, L34D) was conjugated with R-phycoerythrin (R-PE) per the manufacturer’s instructions (Thermo Fisher Scientific, Waltham, MA, USA, S10467) and stored at 4 °C. Antibody titrations were performed for each antibody conjugation to determine each optimal antibody dilution. Following the washing steps of the norovirus–bacterial attachment assay, bacterial pellets were resuspended in 350 uL of blocking buffer (5% BSA powder (*w*/*v*), flow cytometry stain buffer (FCSB; BD Biosciences, Franklin Lakes, NJ, USA; # 554657)) and kept on ice. 50 µL aliquots were mixed 1:1 with conjugated NoV-antibody dilution, the same dilution of isotype control (IgG2b, Invitrogen, Waltham, MA, USA; 12473281) or blocking buffer (negative control). Samples were placed on ice and incubated in the dark for 30 min. After incubation, 100 µL FCSB was added to each sample and the samples centrifuged, 10,000× *g*, 5 min. Pellets were then resuspended in 150 µL FCSB. Samples were transferred in FACS tubes and 400 uL of FCSB was added. Samples were stored for up to 4 h at 4 °C prior to being analyzed on an LSR Fortessa flow cytometer (BD Biosciences, Franklin Lakes, NJ, USA). Using the Overton histogram subtraction method, the fluorescence levels of the antibody-stained samples were compared to the isotype control samples. FCS Express 7 and Graph Pad Prism 8 software were used to analyze samples, *p*-values were determined using Student’s *t*-test.

### 2.6. RT-qPCR Quantification of MNV

Bacterial pellets were resuspended in 600 µL of RNA lysis buffer (Zymo Research, Irvine, CA, USA). Samples were mixed by pipetting and vortexing, and stored at −80 °C. Sample RNA was extracted using the Zymo Research (Irvine, CA, USA) Total RNA Purification procedures from Quick-RNA™ Miniprep Kit. Samples were eluted with 30 µL of nuclease free water at 70 °C. Samples were stored at −80 °C prior to cDNA synthesis (Promega ImProm-II™ Reverse Transcription System). qPCR was performed using PowerTrack SYBR Green Master Mix PCR Kit (Thermo Fisher, Waltham, MA, USA) and 2.5 µL of cDNA per reaction on a QuantStudio™ 5 Real-Time PCR System thermocycler (Thermo Fisher, Walthman, MA, USA) as previously performed [16,29].

## 3. Results

### 3.1. Glycan Microarray

**Figure 1:**
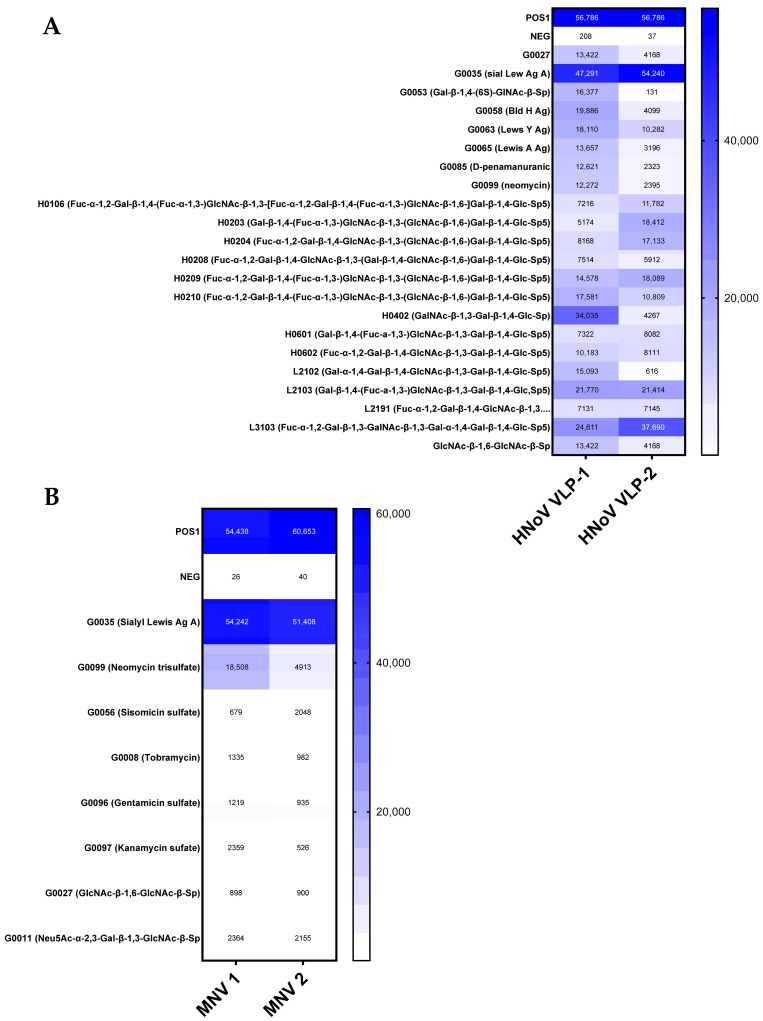
Heat Map of norovirus attachment to select glycan targets. Positive controls were standardized amounts of biotinylated IgG protein printed directly onto the array while negative controls contain only the buffer used to dilute the glycans. Values were normalized to mean signal intensity of positive controls, averaged, and local background signal was subtracted prior to analysis. “1” and “2” represent separate runs using different vials of VLP and virus. POS = Biotynylated IgG protein printed on the array. NEG = buffer diluent used for the carbohydrate targets. (**A**) Targets bound by HuNoV VLPs that averaged signal intensity above 7000 relative signal intensity. (**B**) Targets bound by MNV that averaged signal intensity above 1000 relative signal intensity. (*n* = 2).

The synthetic glycans present in the glycan microarray consisted of four groups: 100 fundamental glycans, 80 N-Glycans, 50 glycolipid glycans, and 50 human milk oligosaccharide glycans. Each of these glycan groups consisted of a diverse variety of other carbohydrates such as monosaccharides, disaccharides, glycosaminoglycans, Lewis Antigens, Blood group glycans, gangliosides, sialylated and natural oligosaccharides, and aminoglycosides (4). Analysis of HuNoV VLP and MNV binding to carbohydrate structures within this glycan microarray demonstrated that both viruses were capable of binding to carbohydrate targets, but to varying degrees (Figure 1). As expected, HuNoV is heavily bound to HBGA and Lewis antigen-containing compounds (Figure 1A). MNV, however, bound selectively to just two bacterial surface structure targets (Sialyl Lews Ag A and Neomycin Trisulfate) with average relative signal intensities greater than 7000 and bound multiple targets with average signal intensities greater than 1000 signal intensity (Figure 1B). Interestingly, many of these targets were aminoglycosides (antibiotics) which share structural similarities with GlcNAc and have been shown to mediate interactions between bacteria and other enteric viruses [8,13]. Both the aminoglycosides and GlcNAc contain amino sugars as core components, feature multiple hydroxyl groups, and contain a six-membered cyclic ring structure, indicating that these components may by key for viral attachment to bacterial cells.

### 3.2. Competitive Inhibition Assays

**Figure 2:**
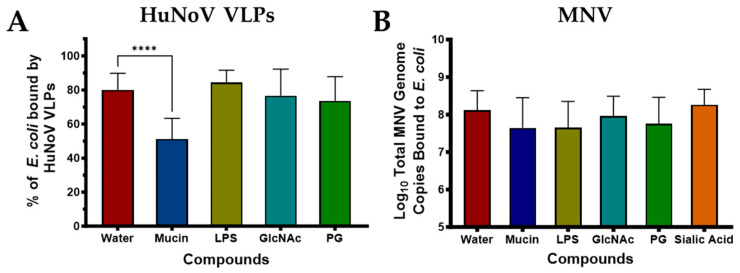
Competitive inhibition assays testing carbohydrate-based targets. The effect of carbohydrate (1000 µg/mL) previously involved in enteric viral attachment on norovirus binding to E. coli BW25113 was assessed for (**A**) HuNoV GII.4 VLPs and (**B**) MNV. *n* = 3–4. **** *p* < 0.0001. LPS = lipopolysaccharide; GlcNAc = N-acetyl-D-glucosamine; PG = peptidoglycan.

In addition to taking a global glycan microarray approach to determine glycan structures that facilitate norovirus binding to commensal bacteria, we also investigated the role of specific carbohydrates in norovirus binding. To do this, we first performed competitive inhibition assays (CIA) using carbohydrates that were previously linked to bacterial binding by enteric viruses [8,11,13,18]. For these assays, either HuNoV VLPs or live MNV were pre-incubated with the compounds prior to use in the bacterial attachment assay, with the idea that if the virus targeted a similar carbohydrate on the bacterial surface, attachment would be reduced since pre-incubation would occupy those sites on the viral capsid. For these experiments, an *E. coli* strain that we have previously shown to have similar levels of viral binding as other commensal bacteria was used [16] (Figure S1). This bacterium was chosen because it is the parent strain of the Keio collection [30] which contains a library of bacteria with mutations in all non-essential genes. Bacterial mutants were included in subsequent experiments to further elucidate structures involved in norovirus–bacterial interactions. Results from CIA assays showed that, as expected for HuNoV, mucin (which contains HBGA like compounds) significantly inhibited viral binding (Figure 2A). Mucin did not eliminate binding which could indicate there are other compounds on the bacterial surface that are bound by HuNoV VLPs or that electrostatic forces also play a role in HuNoV binding to bacteria. Since it has been previously shown that poliovirus (another enteric viral pathogen) bind to LPS and GlcNAc sugars on commensal bacteria, we also assessed whether these were involved in norovirus binding. Pre-incubation with these compounds or peptidoglycan did not reduce HuNoV VLP binding to bacteria (Figure 2A), indicating these carbohydrates are not involved in HuNoV binding.

Like HuNoV, MNV also binds to commensal bacteria, but the specific compounds used by MNV to bind bacteria are unknown. We also performed CIA assays with the same compounds using MNV. Interestingly, although the glycan array data indicated that the virus bound heavily to a specific Lewis antigen, binding of this virus was not inhibited by mucin (Figure 2B). Likewise, other common bacterial structures (LPS and PG) nor sugars found to promote bacterial binding by poliovirus [8,13] led to significant inhibition of MNV. Since sialic acid has been identified as a surface receptor for MNV on host cells [31] and bacteria can express sialic acid like compounds on their surface, we also performed CIA assays using this compound. However, like the other compounds tested, pre-incubation with sialic acid also did not result in reduced viral binding (Figure 2B).

**Figure 3:**
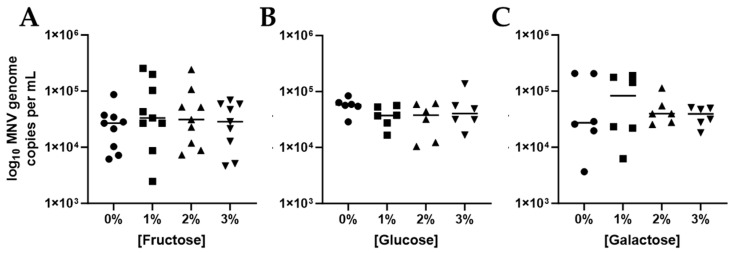
Competitive inhibition assays testing simple sugar inhibition of MNV binding to bacteria. The effect of simple carbohydrates that are often terminal sugars on more complex glycans were tested for their ability to inhibit MNV: bacterial interactions. MNV attachment to *E. coli* BW25113 was assessed for (**A**) Fructose, (**B**) Glucose, and (**C**) Galactose at concentrations ranging from 0 to 3% sugar solution. Each symbol represents a different concentration. *n* = 3–4.

Simpler glycans were also identified in arrays as possible binding targets for MNV. In addition, simple compounds such as fructose, glucose and galactose are often the terminal sugars on more complex glycans. Therefore, we hypothesized that binding to these simpler complexes would support the diversity of low level MNV binding that was observed with the glycan arrays. To test this hypothesis, we repeated our CIA assays using various concentrations of fructose, glucose and galactose. Neither fructose nor glucose incubation led to changes in MNV attachment to *E. coli* (Figure 3A,B). Pre-incubation with galactose did display an average increase in MNV attachment but this change was not significant and was not observed even with higher galactose concentrations (Figure 3C). These and the remaining experiments outlined herein were only performed using MNV. After performing the glycan microarray and initial CIA experiments with HuNoV VLPs, the VLP manufacturer shifted to generating VLPs using the baculovirus system from the human cell-based system they previously used. Unfortunately, VLPs generated using baculovirus did not bind to our commensal bacteria. While not explored further in this manuscript, this indicates that the expression system used to generate HuNoV VLPs changes their external features in such a way that alters VLP binding patterns.

### 3.3. Role of Fucose in MNV Binding to E. coli

**Figure 4:**
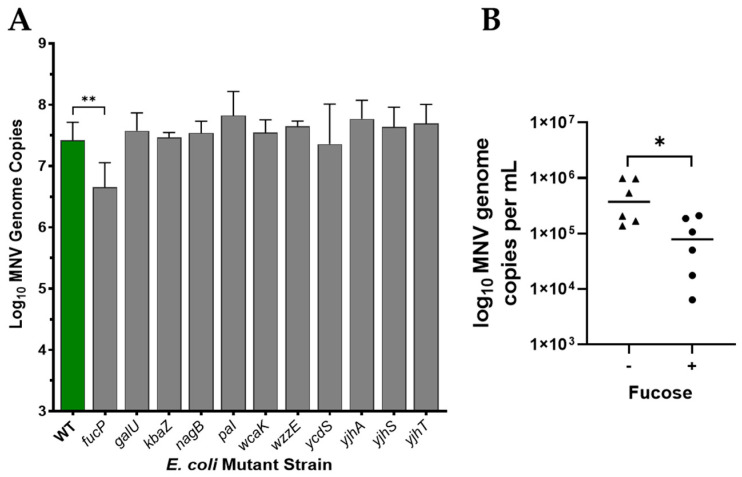
Assessing MNV attachment to select *E. coli* Keio strains containing single-gene deletions in key carbohydrate targets. (**A**) Attachment assays using MNV and each bacterial strain were performed in parallel alongside the wild-type strain. (**B**) CIA assays incubated MNV with 1 mg/mL of fucose for 1 hr prior to performing the attachment assay with *E. coli.* MNV genomes were subsequently quantified using RT-qPCR where the symbols represent the different sugar concentrations. Statistical significance was analyzed using Student’s *t*-test in GraphPad Prism 10, (*n* = 3), * *p* < 0.05, ** *p* ≤ 0.01.

To further examine binding, we selected bacterial mutants missing carbohydrate structures to see if they impacted binding. Mutant bacterial strains with single gene deletions for carbohydrate-based structures or regulation underwent attachment assays with MNV, detailed in Table 1. Relative to the wild-type (WT) strain only deletion of *fucP* resulted in a significant reduction in viral attachment (Figure 4A). All other mutant strains showed similar or slightly higher genome copies compared to the WT, suggesting loss of these structures do not significantly impact MNV attachment to the bacteria. The *fucP* gene mediates cellular uptake of L-fucose, and loss of this gene could possibly reduces cellular fucose concentrations. Although there were no changes in binding for other *fuc* catabolic genes, the reduction in binding to *fucP* led us to test the role of fucose in MNV binding using CIA experiments. Indeed, results showed pre-incubation of MNV with fucose resulted in a significant reduction in viral binding to *E. coli* (Figure 4B). Binding was not eliminated which demonstrates that, while fucose may play a role in MNV: bacterial interactions, it is not the only compound that mediates viral attachment.

### 3.4. Role of Proteinaceous Structures in MNV Binding to E. coli

**Figure 5:**
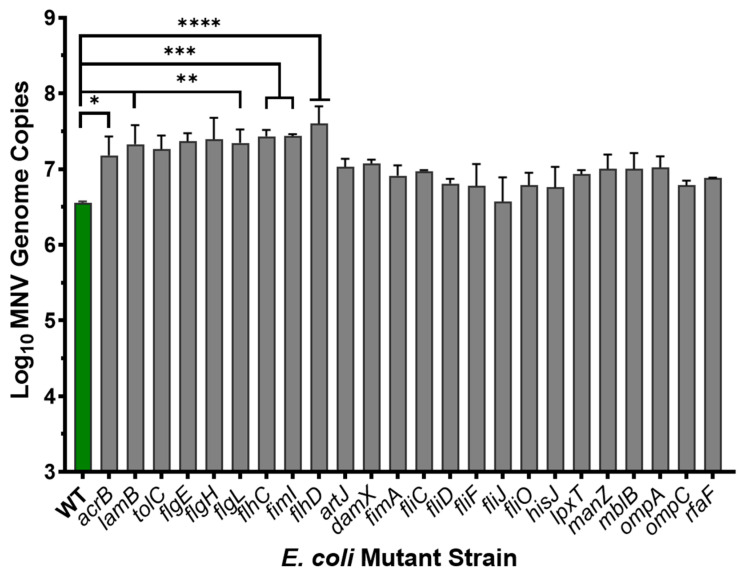
Assessing MNV attachment to select *E. coli* Keio strains containing single-gene deletions in key proteinaceous surface structures. Attachment assays using MNV and each bacterial strain were performed in parallel alongside the wild-type strain. MNV genomes subsequently quantified using RT-qPCR. Statistical significance was analyzed using Student’s *t*-test in GraphPad Prism 10, (*n* = 3), * *p* < 0.05, ** *p* ≤ 0.01, *** *p* ≤ 0.001, **** *p* ≤ 0.0001.

Since it has been previously shown that HuNoV VLPs bound to protein fractions of bacterial lysates containing outer membrane proteins and since limited positive results were obtained with carbohydrate experiments, we again employed the Keio deletion mutant collection to determine the impact of proteinaceous surface structures on MNV: bacterial binding. Interestingly, results showed that loss of expression of several bacterial surface structure including flagella (*flgE*, *flgH, flgL, flhC, fhD*), pillus (*fimI*), and other various outer membrane proteins (*acrB, lamB, tolC*) led to significant increases in MNV attachment, while none of the tested protein mutants led to reduced viral attachment (Figure 5). Loss of flagella in *E. coli* can lead to increased carbohydrate capsule expression as a result of the energy saved due to the lack of flagellar production [32]. Therefore, this result is another piece of indirect evidence pointing to carbohydrate-based structures as the primary target for MNV primarily attachment to bacterial cells.

### 3.5. Binding of MNV Strains to E. coli

**Figure 6:**
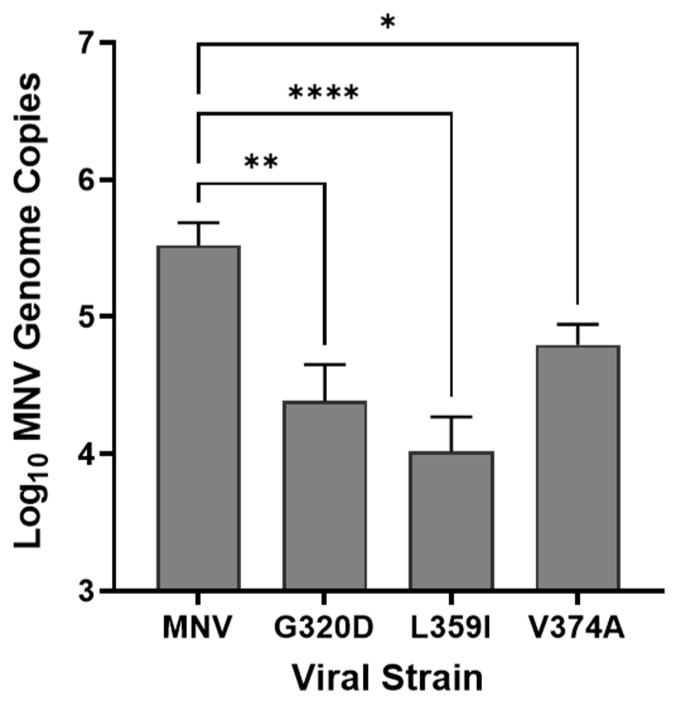
Impact of change in P2 domain residues on MNV attachment to *E. coli.* Attachment assays were performed using MNV mutant viruses and the *E. coli* Keio wild-type strain. Attachment assays with all mutants were performed in parallel with wild-type MNV, and MNV genomes were quantified using RT-qPCR. Statistical significance was analyzed using Student’s *t*-test in GraphPad Prism 10, (*n* = 3), * *p* < 0.05, ** *p* ≤ 0.01, **** *p* ≤ 0.0001.

Investigations into the regions of the MNV capsid that are associated with infection and disease have found that several residues within the P2 domain of VP1 are associated with reduced lethality in IFNAR^−/−^ mice or reduced disease severity in infant mouse models [27,28]. Since interactions with commensal bacteria are also involved in MNV infection, we tested the ability of a small cohort of MNV mutant viruses to attach to *E. coli*. Results showed that all three MNV strains (G320D, L359I, and V374A) were significantly reduced in their ability to attach to the bacteria (Figure 6). While not a definitive link between the ability to bind commensal bacteria and virulence, this data provides a foundation for exploring the importance of the direct MNV: bacterial interaction on the ability of the virus to cause disease.

## 4. Discussion

The findings of this study align with and expand upon existing knowledge of norovirus–bacterial interactions and provide valuable insights into the carbohydrate-mediated interactions between noroviruses and bacterial surfaces. This ultimately contributes to the broader understanding of enteric virus attachment mechanisms which have implications for understanding viral infection dynamics as well as the potential use of bacterial products as therapeutic interventions for norovirus infection. In this study, the glycan microarray data confirmed that both MNV and HuNoV can bind selectively to specific carbohydrate structures. The results indicate that MNV in particular may be capable of selectively binding to a subset of bacterial glycans featuring fucosylated structures, which corroborates earlier findings on glycan-mediated stabilization of viral particles [4]. In contrast, HuNoV exhibited a broader binding preference, including compounds present in mucin and several antibiotic compounds. These findings are consistent with prior studies indicating that enteric viruses exploit host and microbial glycans and glycosylated proteins for attachment and stability [11,13]. For instance, poliovirus has been shown to bind to LPS and GlcNAc moieties, suggesting a common strategy among enteric viruses [8,13].

The competitive inhibition assays further refine this understanding, showing that while certain glycans such as mucin can significantly inhibit HuNoV binding, their efficacy varies across viral strains and expression systems. This variability highlights the complexity of viral recognition of bacterial structures and underscores the importance of glycan diversity in the gut microbiome [11,33]. These results also support previous findings that human histo-blood group antigens (HBGAs) mediate viral attachment [4]. However, the lack of inhibition by LPS, GlcNAc, or sialic acid compounds for both MNV and HuNoV points to the potential involvement of other, yet unidentified, carbohydrate structures. This finding is significant because it challenges the prevailing assumption that LPS or GlcNAc universally mediate viral-bacterial interactions [8,13], indicating that norovirus attachment mechanisms may involve a broader repertoire of bacterial surface structures than previously appreciated and highlights the need for further exploration of bacterial glycans.

The ability of a simple sugar like fucose to inhibit MNV binding, as observed in competitive inhibition assays, is particularly intriguing. While this sugar does not mimic HBGAs, its ability to reduce viral binding highlights the versatility of carbohydrate-virus interactions. It is also possible that electrostatic interactions, as suggested by the positively charged amino groups in aminoglycosides and their similarity to GlcNAc structures, may play a role in this binding process, warranting additional mechanistic studies. The GlcNAc moiety is the most prevalent glycan found on the surface of mammalian cells [34] and the polymeric-glucosamine (p-GlcNAc) moiety has been shown to play a role in biofilm formation of Gram-negative bacteria [35] and attachment of poliovirus to commensal microbiota [8,13] which also points to the significance of the aminoglycoside structure in viral interactions. For example, tobramycin, kanamycin, and neomycin contain glucosamine and related structures and these sugars are often substituted with amino or hydroxyl groups, allowing them to participate in biochemical interactions similar to p-GlcNAcs [36]. In addition, p-GlcNAc and the sugar components of aminoglycosides contain multiple hydroxyl groups which may also be critical for biological interactions [37,38].

Ultimately, the study’s findings have broader implications for understanding the ecological and evolutionary dynamics of noroviruses. Viral attachment to commensal bacteria is thought to enhance stability and facilitate persistence in the gut environment [4,8,10,12,13,20]. However, the observation that MNV stability does not necessarily translate to increased infectivity suggests a nuanced role for bacterial interactions [11], diverging from mechanisms employed by other enteric viruses. From a public health perspective, the antigenic diversity of HuNoVs presents significant challenges for detection and management. With over 30 genotypes and frequent emergence of new variants, current detection methods are insufficiently robust to capture this diversity [39,40]. The identification of sugar-specific binding preferences offers a promising avenue for developing more effective virus capture and detection technologies. Specifically, targeting sugar moieties bound by HuNoVs could lead to advancements in diagnostic tools for food and environmental samples.

## 5. Conclusions

This study underscores the significance of norovirus–bacterial interactions in shaping viral stability, attachment, and host–pathogen dynamics. By identifying both specific and variable glycan targets of HuNoV and MNV, these findings offer valuable insights into the nuanced interplay between noroviruses and bacterial glycans, with implications for understanding viral transmission and persistence in diverse environmental and host settings. As the study of virus–glycan interactions progresses, further research is warranted to explore the broader implications of these norovirus–bacterial interactions, including their relevance to other enteric pathogens and their application in antiviral interventions.

## Figures and Tables

**Table 1:** *E. coli* Keio collection strains used to test MNV attachment.

Reference Name	Gene Deletion	Encoded Product
BW25113	Wild type	N/A
Carbohydrate Mutant Strains		
JW2773	*fucI*	Converts aldose L-fucose to ketose L-fuculose
JW2772	*fucP*	Mediates cellular uptake of L-fucose
JW1224	*galU*	Galactose catabolic process
JW3101	*kbaZ*	Carbohydrate metabolism
JW0664	*nagB*	N-acetylglucosamine degradation
JW0731	*pal*	Peptidoglycan-associated lipoprotein
JW2030	*wcaK*	Biosynthesis of colonic acid
JW5601	*wzzE*	Enterobacterial common antigen assembly
JW1010	*ycdS*	PGA export across the outer membrane
JW5778	*yjhA*	Outer membrane protein for Neu5Ac entry
JW4272	*yjhS*	Catalyzes hydrolysis of alternative sialic acid
JW5777	*yjhT*	Neu5Ac α- and β-anomers conversion
Protein Mutant Strains		
JW0451	*acrB*	Efflux pump membrane transporter
JW0844	*artJ*	Arginine-binding periplasmic protein 2
JW3351	*damX*	Cellular division protein
JW4277	*fimA*	Pilus/cell adhesion
JW4278	*fimI*	Pilus/cell adhesion
JW0940	*flgE*	Flagella hook protein
JW1066	*flgH*	Cytoplasmic flagellar protein
JW1067	*flgI*	Flagellar assembly outer membrane protein
JW1880	*flhC*	Master regulator of flagella assembly
JW1881	*flhD*	Master regulator of flagella assembly
JW1908	*fliC*	Extracellular flagellar component
JW1909	*fliD*	Regulator and cap of flagella
JW1922	*fliF*	Membrane flagellar component
JW1926	*fliJ*	Membrane flagellar component
JW1931	*fliO*	Membrane flagellar component
JW2306	*hisJ*	Outer membrane protein
JW3996	*lamB*	Ion transport outer membrane protein
JW2162	*lpxT*	Involved in modification of Lipid A of LPS
JW1808	*manZ*	Intracellular protein
JW2137	*mglB*	Pilus/cell adhesion protein
JW2203	*ompC*	Ion transport outer membrane protein
JW3595	*rfaF*	Involved in lipopolysaccharide formation
JW3003	*tolC*	Outer membrane TolC type 1 secretion protein

## Data Availability

Data will be made available in the publicly available repository Open Science Framework at osf.io.

## Supplementary Materials

The following supporting information can be downloaded at: https://www.mdpi.com/article/10.3390/v17081142/s1, Table S1: Glycan structures in Chemily Glycan Microarray 300; Figure S1: Human Norovirus GII.4 VLP binding to *E. coli*.
