# Supplementary material for: Determining the Importance of Carbohydrate-Based Structures in Murine Norovirus Binding to Commensal Bacteria
Source: Viruses. 2025 Aug 20;17(8):1142. doi: 10.3390/v17081142 (PMC12390702; doi:10.3390/v17081142)
Supplement: Supplementary file 1 [file viruses-17-01142-s001.zip › viruses-3384304-supplementary.pdf]

Table 1.

Structure	ID
IgG Biotinylated Protein	POS1
Buffer	NEG
β-Glc-Sp	G0001
β-Gal-Sp	G0002
α-Man-Sp	G0003
α-Fuc-Sp	G0004
α-Rha-Sp	G0005
β-GlcNAc-Sp	G0006
β-GalNAc-Sp	G0007
Tobramycin	G0008
Gal-β-1,3-GlcNAc-β-Sp	G0009
Gal-α-1,3-Gal-β-1,3-GlcNAc-β-Sp	G0010
Neu5Ac-α-2,3-Gal-β-1,3-GlcNAc-β-Sp	G0011
Neu5Ac-α-2,6-Gal-β-1,3-GlcNAc-β-Sp	G0012
Neu5Gc-α-2,3-Gal-β-1,3-GlcNAc-β-Sp	G0013
Neu5Gc-α-2,6-Gal-β-1,3-GlcNAc-β-Sp	G0014
Gal-β-1,3-(Fuc-α-1,4)-GlcNAc-β- [Lewis A] –Sp	G0015
Gal-β-1,4-Glc-β-Sp	G0016
Gal-α-1,3-Gal-β-1,4-Glc-β-Sp	G0017
Gal-α-1,4-Gal-β-1,4-Glc-β-Sp	G0018
GlcNAc-β-1,3-Gal-β-1,4-Glc-β-Sp	G0019
GalNAc-β-1,3-Gal-β-1,4-Glc-β-Sp	G0020
Neu5Ac-α-2,3-Gal-β-1,4-Glc-β-Sp	G0021
Neu5Ac-α-2,6-Gal-β-1,4-Glc-β-Sp	G0022
Neu5Gc-α-2,3-Gal-β-1,4-Glc-β-Sp	G0023
Neu5Ac-α-2,6-Gal-β-1,4-Glc-β-Sp	G0024

Gal-β-1,4-(Fuc-α-1,3)-Glc-β-Sp	G0025
GalNAc-β-1,3-Gal-α-1,4-Gal-β-1,4-Glc-β-Sp	G0026
GlcNAc-β-1,6-GlcNAc-β-Sp	G0027
4-P-GlcNAc-b-1,4-Man-b-Sp	G0028
Glc-α-1,2-Gal-α-1,3-Glc-α-Sp	G0029
Gal-β-1,3-GalNAc-α-Sp	G0030
Gal-β-1,4-GlcNAc-β-Sp	G0031
Gal-β-1,4 -(Fuc-α-1,3)-GlcNAc-β- [Lewis X] –Sp	G0032
Neu5Ac-α-2,3-Gal-β-1,4-(Fuc-α-1,3)-GlcNAc-β- [Sialyl Lewis X]-Sp	G0033
Neu5Ac-α-2,3-Gal-β-1,3 -(Fuc-α-1,4)-GlcNAc-β- [Sialyl Lewis A]-Sp	G0034
Neu5Gc-α-2,3-Gal-β-1,3-(Fuc-α-1,4)-GlcNAc-β- [Sialyl Lewis A]-Sp	G0035
Gal-α-1,4-Gal-β-1,3-GlcNAc-β-Sp	G0036
Gal-β-1,4-GlcNAc-β-1,3-Gal-β-1,4-Glc-β- [LNnT]-Sp	G0037
GlcA-β-1,4-GlcNAc-α-1,4-GlcA-β-Sp	G0038
GlcNAc-β-1,6-(Gal-β-1,3)-GalNAc-α-O-Ser-Sp4	G0039
Neu5Ac-α-2,3Gal-β-1,4-(6S)GlcNAc-β-Sp	G0040
GalNAc-β-1,4-GlcNAc-β-Sp2	G0041
Neu5Ac-α-2,8-Neu5Ac-α-2,3-Gal β-1,4-Glc-β-Sp	G0042
Neu5Gc-α-2,8-Neu5Ac-α-2,3-Gal-β-1,4-Glc-β-Sp	G0043
GalNAc-α-1,3-(Fuc-α-1,2)-Gal-β-1,4-Glc-β- [Blood A antigen tetrose]-Sp1	G0044
GlcNAc-β-1,2-Man-α-Sp	G0045
Neu5Ac-α-2,3-Gal-β-Sp1	G0046
Gal-β-1,3 -GalNAc-β-1,3-Gal-β-Sp1	G0047
Gal-α-1,2-Gal-α-Sp	G0048
Gal-β-1,4-(Fuc-α-1,3)-GlcNAc-β-1,3-Gal-β-Sp1	G0049
Neu5Ac-α-2,3-Gal-β-1,4-(Fuc-α-1,3)-Glc-β- [3-Sialyl-3-[illegible]ucosyllactose/ F-SL]-Sp1	G0050
GlcNAc-β-1,4-GlcNAc-β-Sp1	G0051
β-D-GlcA-Sp	G0052
Gal-β-1,4-(6S)GlNAc-β-Sp	G0053

GlcNAc-α-1,3-(Glc-α-1,2-Glc-α-1,2)-Gal-α-1,3-Glc-α-Sp	G0054
Gal-β-1,3-GalNAc-β-1,4-(Neu5Gc-α-2,3)-Gal-β-1,4-Glc-β-Sp1	G0055
Sisomicin Sul[illegible]ate	G0056
GalNAc-α-1,3-(Fuc-α-1,2)-Gal-β- [Blood A antigen trisaccharide]-Sp1	G0057
Fuc-α-1,2-Gal-β-1,4-GlcNAc-β- [Blood H antigen trisaccharide]-Sp1	G0058
Gal-α-1,3-(Fuc-α-1,2)-Gal-β- [Blood B antigen trisaccharide]-Sp1	G0059
Fuc-α-1,2-Gal-β-1,3-GlcNAc-β-1,3-Gal-β-1,4-Glc-β- [LNFP I]-Sp1	G0060
Fuc-α-1,2-Gal-β-1,4-Glc-β- [Blood H antigen trisaccharide]-Sp1	G0061
Gal-α-1,3-(Fuc-α-1,2)-Gal-β-1,4-Glc-β- [Blood B antigen tetrasaccharide]-Sp1	G0062
(Fuc-α-1,2)-Gal-β-1,4-(Fuc-α-1,3)-GlcNAc-β- [Lewis Y]-Sp1	G0063
(Fuc-α-1,2)-Gal-β-1,3-(Fuc-α-1,4)-GlcNAc-β- [Lewis B]-Sp1	G0064
Gal-β-1,3-(Fuc-α-1,4)-GlcNAc-β-1,3-Gal-β-1,4-(Fuc-α-1,4)-Glc-β- [Lewis A]-Sp1	G0065
Gal-β-1,3-GalNAc-β-Sp1	G0066
Gal-β-1,3-(Neu5Ac-α-2,6)-GalNAc-β-Sp	G0067
Neu5Ac-α-2,6-Gal-β-1,3-GalNAc-β-Sp	G0068
Neu5Ac-α-2,6-Gal-β-1,3-(Neu5Ac-α-2,6)-GalNAc-β-Sp	G0069
Neu5Ac-α-2,3-Gal-β-1,3-(Neu5Ac-α-2,6)-GalNAc-β-Sp	G0070
Neu5Ac-α-2,6-(Neu5Ac-α-2,3)-Gal-β-1,3-GalNAc-β-Sp	G0071
GalNAc-β-1,4-(Neu5Ac-α-2,3)-Gal-β-1,4-Glc-β- [GM2]-Sp	G0072
GalNAc-β-1,4-(Neu5Ac-α-2,8-Neu5Ac-α-2,3)-Gal-β-1,4-Glc-β- [GD2]-Sp	G0073
Gal-α-1,4-Gal-β-1,4-GlcNAc-β-Sp1	G0074
β-D-Rha-Sp	G0075
Glc-α-1,4-Glc-β-Sp1	G0076
Glc-α-1,6-Glc-α-1,4-Glc-β-Sp1	G0077
Maltotriose-β-Sp1	G0078
Glc-α-1,6-Glc-α-1,6-Glc-β-Sp1	G0079
Maltotetraose-β-Sp1	G0080
GlcNAc-α-1,4-GlcA-β-1,4-GlcNAc-α1,4-GlcA-β-Sp	G0081
Maltohexaose-β-Sp1	G0082

Maltoheptaose-β-Sp1	G0083
Acarbose-β-Sp1	G0084
D-pentamannuronic acid-β-Sp1	G0085
L-pentaguluronic acid-β-Sp1	G0086
D-cellose-β-Sp1	G0087
Gal-α-1,3-Gal-β-Sp1	G0088
β-1,4-Xylotetrose-Sp1	G0089
Chitin-trisaccharide-Sp1	G0090
KDN-α-2,8-Neu5Ac-α-2,3-Gal-β-1,4-Glc-β-Sp	G0091
Neu5Ac-α-2,8-Neu5Gc-α-2,3-Gal-β-1,4-Glc-β-Sp	G0092
Neu5Ac-α-2,8-Neu5Ac-α-2,8-Neu5Ac-α-2,3-Gal-β-1,4-Glc-β-Sp3	G0093
Neu5Ac-a-2,8-Neu5Ac-a-2,6-Gal-b-1,4-Glc-Sp5	G0094
Gal-β-1,3-GalNAc-β-1,4-(Neu5Ac-α-2,3)-Gal-β-1,4-Glc-β-Sp1	G0095
Gentamicin Sul[illegible]ate	G0096
Kanamycin sul[illegible]ate	G0097
Geneticin Disul[illegible]ate Salt (G418)	G0098
Neomycin trisul[illegible]ate	G0099
SGP	G0100
Man-α-1,6-(Man-α-1,3-)Man-α-1,6-(GlcNAc-β-1,2-Man-α-1,3-)Man-β-1,4-GlcNAc-β-1,4-GlcNAc-Sp5	N010
Man-α-1,6-(Man-α-1,3-)Man-α-1,6-(Gal-β-1,4-GlcNAc-β-1,2-Man-α-1,3-)Man-β-1,4-GlcNAc-β-1,4-GlcNAc-Sp5	N011
Man-α-1,6-(Man-α-1,3-)Man-α-1,6-(Neu5Ac-α-2,3-Gal-β-1,4-GlcNAc-β-1,2-Man-α-1,3-) Man-β-1,4-GlcNAc-β-1,4-GlcNAc-Sp5	N012
Man-α-1,6-(Man-α-1,3-)Man-α-1,6-(Neu5Ac-α-2,6-Gal-β-1,4-GlcNAc-β-1,2-Man-α-1,3-) Man-β-1,4-GlcNAc-β-1,4-GlcNAc-Sp5	N013
Man-α-1,6-(Man-α-1,3-)Man-α-1,6-[Gal-β-1,4-(Fuc-α-1,3-)GlcNAc-β-1,2-Man-α-1,3-]Man-β-1,4-GlcNAc-β-1,4-GlcNAc-Sp5	N014
Man-α-1,6-(Man-α-1,3-)Man-α-1,6-[Neu5Ac-α-2,3-Gal-β-1,4-(Fuc-α-1,3-)GlcNAc-β-1,2- Man-α-1,3-]Man-β-1,4-GlcNAc-β-1,4-GlcNAc-Sp5	N015
GlcNAc-β-1,2-Man-α-1,3-Man-β-1,4-GlcNAc-β-1,4-GlcNAc-Sp5	N020

Gal-β-1,4-GlcNAc-β-1,2-Man-α-1,3-Man-β-1,4-GlcNAc-β-1,4-GlcNAc-Sp5	N021
Neu5Ac-α-2,3-Gal-β-1,4-GlcNAc-β-1,2-Man-α-1,3-Man-β-1,4-GlcNAc-β-1,4-GlcNAc-Sp5	N022
Neu5Ac-α-2,6-Gal-β-1,4-GlcNAc-β-1,2-Man-α-1,3-Man-β-1,4-GlcNAc-β-1,4-GlcNAc-Sp5	N023
Gal-β-1,4-(Fuc-α-1,3-)GlcNAc-β-1,2-Man-α-1,3-Man-β-1,4-GlcNAc-β-1,4-GlcNAc-Sp5	N024
Neu5Ac-α-2,3-Gal-β-1,4-(Fuc-α-1,3-)GlcNAc-β-1,2-Man-α-1,3-Man-β-1,4-GlcNAc-β-1,4-GlcNAc-Sp5	N025
Neu5Gc-α-2,3-Gal-β-1,4-GlcNAc-β-1,2-Man-α-1,3-Man-β-1,4-GlcNAc-β-1,4-GlcNAc-Sp5	N026
Neu5Gc-α-2,6-Gal-β-1,4-GlcNAc-β-1,2-Man-α-1,3-Man-β-1,4-GlcNAc-β-1,4-GlcNAc-Sp5	N022G
Neu5Gc-α-2,3-Gal-β-1,4-(Fuc-α-1,3-)GlcNAc-β-1,2-Man-α-1,3-Man-β-1,4-GlcNAc-β-1,4-GlcNAc-Sp5	N023G
Gal-α-1,3-Gal-β-1,4-GlcNAc-β-1,2-Man-α-1,3-Man-β-1,4-GlcNAc-β-1,4-GlcNAc-Sp5	N025G
Man-α-1,6-(GlcNAc-β-1,2-Man-α-1,3-)Man-β-1,4-GlcNAc-β-1,4-GlcNAc-Sp5	N030
GlcNAc-β-1,2-Man-α-1,6-[GlcNAc(3Ac)-β-1,2-Man-α-1,3-Man-β-1,4-GlcNAc-β-1,4-GlcNAc-Sp5	N210
GlcNAc-β-1,2-Man-α-1,6-Man-β-1,4-GlcNAc-β-1,4-GlcNAc-Sp5	N040
Gal-β-1,4-GlcNAc-β-1,2-Man-α-1,3-Man-β-1,4-GlcNAc-β-1,4-GlcNAc-Sp5	N041
Neu5Ac-α-2,3-Gal-β-1,4-GlcNAc-β-1,2-Man-α-1,3-Man-β-1,4-GlcNAc-β-1,4-GlcNAc-Sp5	N042
Neu5Ac-α-2,6-Gal-β-1,4-GlcNAc-β-1,2-Man-α-1,3-Man-β-1,4-GlcNAc-β-1,4-GlcNAc-Sp5	N043
Gal-β-1,4-(Fuc-α-1,3-)GlcNAc-β-1,2-Man-α-1,3-Man-β-1,4-GlcNAc-β-1,4-GlcNAc-Sp5	N044
Neu5Ac-α-2,3-Gal-β-1,4-(Fuc-α-1,3-)GlcNAc-β-1,2-Man-α-1,3-Man-β-1,4-GlcNAc-β-1,4-GlcNAc-Sp5	N045
GlcNAc-β-1,2-Man-α-1,6-(Man-α-1,3-)Man-β-1,4-GlcNAc-β-1,4-GlcNAc-Sp5	N050
Gal-β-1,4-GlcNAc-β-1,2-Man-α-1,6-(Man-α-1,3-)Man-β-1,4-GlcNAc-β-1,4-GlcNAc-Sp5	N051
Neu5Ac-α-2,3-Gal-β-1,4-GlcNAc-β-1,2-Man-α-1,6-(Man-α-1,3-)Man-β-1,4-GlcNAc-β-1,4-GlcNAc-Sp5	N052
Neu5Ac-α-2,6-Gal-β-1,4-GlcNAc-β-1,2-Man-α-1,6-(Man-α-1,3-)Man-β-1,4-GlcNAc-β-1,4-GlcNAc-Sp	N053
Gal-β-1,4-(Fuc-α-1,3-)GlcNAc-β-1,2-Man-α-1,6-(Man-α-1,3-)Man-β-1,4-GlcNAc-β-1,4-GlcNAc-Sp5	N054
Neu5Ac-α-2,3-Gal-β-1,4-(Fuc-α-1,3-)GlcNAc-β-1,2-Man-α-1,6-(Man-α-1,3-)Man-β-1,4-GlcNAc-β-1,4-GlcNAc-Sp5	N055
Neu5Ac-α-2,6-Gal-β-1,4-GlcNAc-Man-α-1,3-(Neu5Ac-α-2,6-Gal-β-1,4-GlcNAc-Man-α-1,6-)Man-β-1,4-GlcNAc-β-1,4-GlcNAc-β-Asn	TE001

Gal-β-1,4-GlcNAc-β-1,2-Man-α-1,3-(Gal-β-1,4-GlcNAc-β-1,2-Man-α-1,6-)Man-β-1,4-GlcNAc-β-1,4-GlcNAc-β-Asn	TE002
Neu5Gc-α-2,6-Gal-β-1,4-GlcNAc-β-1,2-Man-α-1,3-(Neu5Gc-α-2,6-Gal-β-1,4-GlcNAc-β-1,2-Man-α-1,6-)Man-β-1,4-GlcNAc-β-1,4-GlcNAc-β-Asn	TE003
Neu5Ac-α-2,3-Gal-β-1,4-GlcNAc-β-1,2-Man-α-1,3-(Neu5Ac-α-2,3-Gal-β-1,4-GlcNAc-β-1,2-Man-α-1,6-)Man-β-1,4-GlcNAc-β-1,4-GlcNAc-β-Asn	TE004
Neu5Gc-α-2,3-Gal-β-1,4-GlcNAc-β-1,2-Man-α-1,3-(Neu5Gc-α-2,3-Gal-β-1,4-GlcNAc-β-1,2-Man-α-1,6-)Man-β-1,4-GlcNAc-β-1,4-GlcNAc-β-Asn	TE005
Gal-β-1,4-(Fucα-1,3-)GlcNAc-β-1,2-Man-α-1,3-[Gal-β-1,4-(Fucα-1,3-)GlcNAc-β-1,2-Man-α-1,6-]Man-β-1,4-GlcNAc-β-1,4-GlcNAc-β-Asn	TE006
Gal-α-1,3-Gal-β-1,4-GlcNAc-β-1,2-Man-α-1,3-(Gal-α-1,3-Gal-β-1,4-GlcNAc-β-1,2-Man-α-1,6-)Man-β-1,4-GlcNAc-β-1,4-GlcNAc-β-Asn	TE007
Gal-β-1,4-(Fucα-1,3-)GlcNAc-β-1,2-Man-α-1,3-[Gal-β-1,4-(Fucα-1,3-)GlcNAc-β-1,2-Man-α-1,6-]Man-β-1,4-GlcNAc-β-1,4-GlcNAc-β-Asn	TE008
Neu5Ac-α-2,8-Neu5Ac-α-2,6-Gal-β-1,4-GlcNAc-β-1,2-Man-α-1,3-(Neu5Ac-α-2,8-Neu5Ac-α-2,6-Gal-β-1,4-GlcNAc-β-1,2-Man-α-1,6-)Man-β-1,4-GlcNAc-β-1,4-GlcNAc-β-Asn	TE009
Neu5Gc-α-2,8-Neu5Ac-α-2,6-Gal-β-1,4-GlcNAc-β-1,2-Man-α-1,3-(Neu5Gc-α-2,8-Neu5Ac-α-2,6-Gal-β-1,4-GlcNAc-β-1,2-Man-α-1,6-)Man-β-1,4-GlcNAc-β-1,4-GlcNAc-β-Asn	TE010
Neu5Ac-α-2,8-Neu5Gc-α-2,6-Gal-β-1,4-GlcNAc-β-1,2-Man-α-1,3-(Neu5Ac-α-2,8-Neu5Gc-α-2,6-Gal-β-1,4-GlcNAc-β-1,2-Man-α-1,6-)Man-β-1,4-GlcNAc-β-1,4-GlcNAc-β-Asn	TE011
Neu5Gc-α-2,8-Neu5Gc-α-2,6-Gal-β-1,4-GlcNAc-β-1,2-Man-α-1,3-(Neu5Gc-α-2,8-Neu5Gc-α-2,6-Gal-β-1,4-GlcNAc-β-1,2-Man-α-1,6-)Man-β-1,4-GlcNAc-β-1,4-GlcNAc-β-Asn	TE012
Neu5Ac-α-2,8-Neu5Ac-α-2,3-Gal-β-1,4-GlcNAc-β-1,2-Man-α-1,3-(Neu5Ac-α-2,8-Neu5Ac-α-2,3-Gal-β-1,4-GlcNAc-β-1,2-Man-α-1,6-)Man-β-1,4-GlcNAc-β-1,4-GlcNAc-β-Asn	TE013
Neu5Gc-α-2,8-Neu5Ac-α-2,3-Gal-β-1,4-GlcNAc-β-1,2-Man-α-1,3-(Neu5Gc-α-2,8-Neu5Ac-α-2,3-Gal-β-1,4-GlcNAc-β-1,2-Man-α-1,6-)Man-β-1,4-GlcNAc-β-1,4-GlcNAc-β-Asn	TE014
Neu5Ac-α-2,8-Neu5Gc-α-2,3-Gal-β-1,4-GlcNAc-β-1,2-Man-α-1,3-(Neu5Ac-α-2,8-Neu5Gc-α-2,3-Gal-β-1,4-GlcNAc-β-1,2-Man-α-1,6-)Man-β-1,4-GlcNAc-β-1,4-GlcNAc-β-Asn	TE015
Neu5Gc-α-2,8-Neu5Gc-α-2,3-Gal-β-1,4-GlcNAc-β-1,2-Man-α-1,3-(Neu5Gc-α-2,8-Neu5Gc-α-2,3-Gal-β-1,4-GlcNAc-β-1,2-Man-α-1,6-)Man-β-1,4-GlcNAc-β-1,4-GlcNAc-β-Asn	TE016
Neu5Ac-α-2,3-Gal-β-1,4-(Fucα-1,3-)GlcNAc-β-1,2-Man-α-1,3-[Neu5Ac-α-2,3-Gal-β-1,4-(Fucα-1,3-)GlcNAc-β-1,2-Man-α-1,6-]Man-β-1,4-GlcNAc-β-1,4-GlcNAc-β-Asn	TE017
Neu5Gc-α-2,3-Gal-β-1,4-(Fucα-1,3-)GlcNAc-β-1,2-Man-α-1,3-[Neu5Gc-α-2,3-Gal-β-1,4-(Fucα-1,3-)GlcNAc-β-1,2-Man-α-1,6-]Man-β-1,4-GlcNAc-β-1,4-GlcNAc-β-Asn	TE018

Neu5Ac-α-2,3-Gal-β-1,4-GlcNAc-β-1,3-Gal-β-1,4-GlcNAc-β-1,2-Man-α-1,3-(Neu5Ac-α-2,3-Gal-β-1,4-GlcNAc-β-1,3-Gal-β-1,4-GlcNAc-β-1,2-Man-α-1,6-)Man-β-1,4-GlcNAc-β-1,4-GlcNAc-β-Asn	TE019
Neu5Gc-α-2,3-Gal-β-1,4-GlcNAc-β-1,3-Gal-β-1,4-GlcNAc-β-1,2-Man-α-1,3-(Neu5Gc-α-2,3-Gal-β-1,4-GlcNAc-β-1,3-Gal-β-1,4-GlcNAc-β-1,2-Man-α-1,6-)Man-β-1,4-GlcNAc-β-1,4-GlcNAc-β-Asn	TE020
Neu5Ac-α-2,6-Gal-β-1,4-GlcNAc-β-1,3-(Neu5AC-α-2,6-)Gal-β-1,4-GlcNAc-β-1,2-Man-α-1,3-[Neu5Ac-α-2,6-Gal-β-1,4-GlcNAc-β-1,3-(Neu5AC-α-2,6-)Gal-β-1,4-GlcNAc-β-1,2-Man-α-1,6-]Man-β-1,4-GlcNAc-β-1,4-GlcNAc-β-Asn	TE021
Neu5Gc-α-2,6-Gal-β-1,4-GlcNAc-β-1,3-(Neu5GC-α-2,6-)Gal-β-1,4-GlcNAc-β-1,2-Man-α-1,3-[Neu5Gc-α-2,6-Gal-β-1,4-GlcNAc-β-1,3-(Neu5GC-α-2,6-)Gal-β-1,4-GlcNAc-β-1,2-Man-α-1,6-]Man-β-1,4-GlcNAc-β-1,4-GlcNAc-β-Asn	TE022
Neu5Ac-α-2,3-Gal-β-1,4-GlcNAc-β-1,3-(Neu5AC-α-2,6-)Gal-β-1,4-GlcNAc-β-1,2-Man-α-1,3-[Neu5Ac-α-2,3-Gal-β-1,4-GlcNAc-β-1,3-(Neu5AC-α-2,6-)Gal-β-1,4-GlcNAc-β-1,2-Man-α-1,6-]Man-β-1,4-GlcNAc-β-1,4-GlcNAc-β-Asn	TE023
GlcNAc-β-1,3-Gal-β-1,4-GlcNAc-β-1,2-Man-α-1,3-(GlcNAc-β-1,3-Gal-β-1,4-GlcNAc-β-1,2-Man-α-1,6-)Man-β-1,4-GlcNAc-β-1,4-GlcNAc-β-Asn	TE024
Gal-β-1,4-GlcNAc-β-1,3-Gal-β-1,4-GlcNAc-β-1,2-Man-α-1,3-(Gal-β-1,4-GlcNAc-β-1,3-Gal-β-1,4-GlcNAc-β-1,2-Man-α-1,6-)Man-β-1,4-GlcNAc-β-1,4-GlcNAc-β-Asn	TE025
GlcNAc-β-1,3-Gal-β-1,4-GlcNAc-β-1,3-Gal-β-1,4-GlcNAc-β-1,2-Man-α-1,3-(GlcNAc-β-1,3-Gal-β-1,4-GlcNAc-β-1,3-Gal-β-1,4-GlcNAc-β-1,2-Man-α-1,6-)Man-β-1,4-GlcNAc-β-1,4-GlcNAc-β-Asn	TE026
Gal-β-1,4-GlcNAc-β-1,3-Gal-β-1,4-GlcNAc-β-1,3-Gal-β-1,4-GlcNAc-β-1,2-Man-α-1,3-(Gal-β-1,4-GlcNAc-β-1,3-Gal-β-1,4-GlcNAc-β-1,3-Gal-β-1,4-GlcNAc-β-1,2-Man-α-1,6-)Man-β-1,4-GlcNAc-β-1,4-GlcNAc-β-Asn	TE027
GlcNAc-β-1,3-Gal-β-1,4-GlcNAc-β-1,3-Gal-β-1,4-GlcNAc-β-1,3-Gal-β-1,4-GlcNAc-β-1,2-Man-α-1,3-(GlcNAc-β-1,3-Gal-β-1,4-GlcNAc-β-1,3-Gal-β-1,4-GlcNAc-β-1,3-Gal-β-1,4-GlcNAc-β-1,2-Man-α-1,6-)Man-β-1,4-GlcNAc-β-1,4-GlcNAc-β-Asn	TE028
Gal-β-1,4-GlcNAc-β-1,3-Gal-β-1,4-GlcNAc-β-1,3-Gal-β-1,4-GlcNAc-β-1,3-Gal-β-1,4-GlcNAc-β-1,2-Man-α-1,3-(Gal-β-1,4-GlcNAc-β-1,3-Gal-β-1,4-GlcNAc-β-1,3-Gal-β-1,4-GlcNAc-β-1,3-Gal-β-1,4-GlcNAc-β-1,2-Man-α-1,6-)Man-β-1,4-GlcNAc-β-1,4-GlcNAc-β-Asn	TE029
GlcNAc-β-1,3-Gal-β-1,4-GlcNAc-β-1,3-Gal-β-1,4-GlcNAc-β-1,3-Gal-β-1,4-GlcNAc-β-1,3-Gal-β-1,4-GlcNAc-β-1,2-Man-α-1,3-(GlcNAc-β-1,3-Gal-β-1,4-GlcNAc-β-1,3-Gal-β-1,4-GlcNAc-β-1,3-Gal-β-1,4-GlcNAc-β-1,3-Gal-β-1,4-GlcNAc-β-1,2-Man-α-1,6-)Man-β-1,4-GlcNAc-β-1,4-GlcNAc-β-Asn	TE030
Gal-β-1,4-GlcNAc-β-1,3-Gal-β-1,4-GlcNAc-β-1,3-Gal-β-1,4-GlcNAc-β-1,3-Gal-β-1,4-GlcNAc-β-1,3-Gal-β-1,4-GlcNAc-β-1,2-Man-α-1,3-(Gal-β-1,4-GlcNAc-β-1,3-Gal-β-1,4-GlcNAc-β-1,3-Gal-β-1,4-GlcNAc-β-1,3-Gal-β-1,4-GlcNAc-β-1,3-Gal-β-1,4-GlcNAc-β-1,2-Man-α-1,6-)Man-β-1,4-GlcNAc-β-1,4- GlcNAc-β-Asn	TE031
GlcNAc-β-1,3-Gal-β-1,4-(Fucα-1,3-)GlcNAc-β-1,2-Man-α-1,3-[GlcNAc-β-1,3-Gal-β-1,4-(Fucα-1,3-)GlcNAc-β-1,2-Man-α-1,6-]Man-β-1,4-GlcNAc-β-1,4-GlcNAc-β-Asn	TE032

Gal-β-1,4-GlcNAc-β-1,3-Gal-β-1,4-(Fucα-1,3-)GlcNAc-β-1,2-Man-α-1,3-[Gal-β-1,4-GlcNAc-β-1,3-Gal-β-1,4-(Fucα-1,3-)GlcNAc-β-1,2-Man-α-1,6-]Man-β-1,4-GlcNAc-β-1,4-GlcNAc-β-Asn	TE033
Neu5Ac-α-2,3-Gal-β-1,4-GlcNAc-β-1,3-Gal-β-1,4-(Fucα-1,3-)GlcNAc-β-1,2-Man-α-1,3-[Neu5Ac-α-2,3-Gal-β-1,4-GlcNAc-β-1,3-Gal-β-1,4-(Fucα-1,3-)GlcNAc-β-1,2-Man-α-1,6-]Man-β-1,4-GlcNAc-β-1,4-GlcNAc-β-Asn	TE034
GlcNAc-β-1,3-Gal-β-1,4-GlcNAc-β-1,3-Gal-β-1,4-(Fucα-1,3-)GlcNAc-β-1,2-Man-α-1,3-[GlcNAc-β-1,3-Gal-β-1,4-GlcNAc-β-1,3-Gal-β-1,4-(Fucα-1,3-)GlcNAc-β-1,2-Man-α-1,6-]Man-β-1,4-GlcNAc-β-1,4-GlcNAc-β-Asn	TE035
Gal-β-1,4-GlcNAc-β-1,3-Gal-β-1,4-GlcNAc-β-1,3-Gal-β-1,4-(Fucα-1,3-)GlcNAc-β-1,2-Man-α-1,3-[Gal-β-1,4-GlcNAc-β-1,3-Gal-β-1,4-GlcNAc-β-1,3-Gal-β-1,4-(Fucα-1,3-)GlcNAc-β-1,2-Man-α-1,6-]Man-β-1,4-GlcNAc-β-1,4-GlcNAc-β-Asn	TE036
GlcNAc-β-1,3-Gal-β-1,4-GlcNAc-β-1,3-Gal-β-1,4-GlcNAc-β-1,3-Gal-β-1,4-(Fucα-1,3-)GlcNAc-β-1,2-Man-α-1,3-[GlcNAc-β-1,3-Gal-β-1,4-GlcNAc-β-1,3-Gal-β-1,4-GlcNAc-β-1,3-Gal-β-1,4-(Fucα-1,3-)GlcNAc-β-1,2-Man-α-1,6-]Man-β-1,4-GlcNAc-β-1,4-GlcNAc-β-Asn	TE037
Gal-β-1,4-GlcNAc-β-1,3-Gal-β-1,4-GlcNAc-β-1,3-Gal-β-1,4-GlcNAc-β-1,3-Gal-β-1,4-(Fucα-1,3-)GlcNAc-β-1,2-Man-α-1,3-[Gal-β-1,4-GlcNAc-β-1,3-Gal-β-1,4-GlcNAc-β-1,3-Gal-β-1,4-GlcNAc-β-1,3-Gal-β-1,4-(Fucα-1,3-)GlcNAc-β-1,2-Man-α-1,6-]Man-β-1,4-GlcNAc-β-1,4-GlcNAc-β-Asn	TE038
Gal-β-1,4-(Fucα-1,3-)GlcNAc-β-1,3-Gal-β-1,4-(Fucα-1,3-)GlcNAc-β-1,2-Man-α-1,3-[Gal-β-1,4-(Fucα-1,3-)GlcNAc-β-1,3-Gal-β-1,4-(Fucα-1,3-)GlcNAc-β-1,2-Man-α-1,6-]Man-β-1,4-GlcNAc-β-1,4-GlcNAc-β-Asn	TE039
GlcNAc-β-1,3-Gal-β-1,4-(Fucα-1,3-)GlcNAc-β-1,3-Gal-β-1,4-(Fucα-1,3-)GlcNAc-β-1,2-Man-α-1,3-[GlcNAc-β-1,3-Gal-β-1,4-(Fucα-1,3-)GlcNAc-β-1,3-Gal-β-1,4-(Fucα-1,3-)GlcNAc-β-1,2-Man-α-1,6-]Man-β-1,4-GlcNAc-β-1,4-GlcNAc-β-Asn	TE040
Gal-β-1,4-GlcNAc-β-1,3-Gal-β-1,4-(Fucα-1,3-)GlcNAc-β-1,3-Gal-β-1,4-(Fucα-1,3-)GlcNAc-β-1,2-Man-α-1,3-[Gal-β-1,4-GlcNAc-β-1,3-Gal-β-1,4-(Fucα-1,3-)GlcNAc-β-1,3-Gal-β-1,4-(Fucα-1,3-)GlcNAc-β-1,2-Man-α-1,6-]Man-β-1,4-GlcNAc-β-1,4-GlcNAc-β-Asn	TE041
Gal-β-1,4-(Fucα-1,3-)GlcNAc-β-1,3-Gal-β-1,4-(Fucα-1,3-)GlcNAc-β-1,3-Gal-β-1,4-(Fucα-1,3-)GlcNAc-β-1,2-Man-α-1,3-[Gal-β-1,4-(Fucα-1,3-)GlcNAc-β-1,3-Gal-β-1,4-(Fucα-1,3-)GlcNAc-β-1,3-Gal-β-1,4-(Fucα-1,3-)GlcNAc-β-1,2-Man-α-1,6-]Man-β-1,4-GlcNAc-β-1,4-GlcNAc-β-Asn	TE042
Gal-α-1,3-Gal-β-1,4-GlcNAc-β-1,3-Gal-β-1,4-GlcNAc-β-1,2-Man-α-1,3-(Gal-α-1,3-Gal-β-1,4-GlcNAc-β-1,3-Gal-β-1,4-GlcNAc-β-1,2-Man-α-1,6-)Man-β-1,4-GlcNAc-β-1,4-GlcNAc-β-Asn	TE043
Gal-α-1,3-Gal-β-1,4-GlcNAc-β-1,3-Gal-β-1,4-(Fucα-1,3-)GlcNAc-β-1,2-Man-α-1,3-[Gal-α-1,3-Gal-β-1,4-GlcNAc-β-1,3-Gal-β-1,4-(Fucα-1,3-)GlcNAc-β-1,2-Man-α-1,6-]Man-β-1,4-GlcNAc-β-1,4-GlcNAc-β-Asn	TE044
Gal-α-1,3-Gal-β-1,4-(Fucα-1,3-)GlcNAc-β-1,3-Gal-β-1,4-(Fucα-1,3-)GlcNAc-β-1,2-Man-α-1,3-[Gal-α-1,3-Gal-β-1,4-(Fucα-1,3-)GlcNAc-β-1,3-Gal-β-1,4-(Fucα-1,3-)GlcNAc-β-1,2-Man-α-1,6-]Man-β-1,4-GlcNAc-β-1,4-GlcNAc-β-Asn	TE045

Gal-α-1,3-Gal-β-1,4-GlcNAc-β-1,3-Gal-β-1,4-GlcNAc-β-1,3-Gal-β-1,4-GlcNAc-β-1,2-Man-α-1,3-(Gal-α-1,3-Gal-β-1,4-GlcNAc-β-1,3-Gal-β-1,4-GlcNAc-β-1,3-Gal-β-1,4-GlcNAc-β-1,2-Man-α-1,6-)Man-β-1,4-GlcNAc-β-1,4-GlcNAc-β-Asn	TE046
Gal-α-1,3-Gal-β-1,4-GlcNAc-β-1,3-Gal-β-1,4-GlcNAc-β-1,3-Gal-β-1,4-(Fucα-1,3-)GlcNAc-β-1,2-Man-α-1,3-[Gal-α-1,3-Gal-β-1,4-GlcNAc-β-1,3-Gal-β-1,4-GlcNAc-β-1,3-Gal-β-1,4-(Fucα-1,3-)GlcNAc-β-1,2-Man-α-1,6-]Man-β-1,4-GlcNAc-β-1,4-GlcNAc-β-Asn	TE047
Gal-α-1,3-Gal-β-1,4-GlcNAc-β-1,3-Gal-β-1,4-(Fucα-1,3-)GlcNAc-β-1,3-Gal-β-1,4-(Fucα-1,3-)GlcNAc-β-1,2-Man-α-1,3-[Gal-α-1,3-Gal-β-1,4-GlcNAc-β-1,3-Gal-β-1,4-(Fucα-1,3-)GlcNAc-β-1,3-Gal-β-1,4-(Fucα-1,3-)GlcNAc-β-1,2-Man-α-1,6-]Man-β-1,4-GlcNAc-β-1,4-GlcNAc-β-Asn	TE048
Gal-α-1,3-Gal-β-1,4-(Fucα-1,3-)GlcNAc-β-1,3-Gal-β-1,4-(Fucα-1,3-)GlcNAc-β-1,3-Gal-β-1,4-(Fucα-1,3-)GlcNAc-β-1,2-Man-α-1,3-[Gal-α-1,3-Gal-β-1,4-(Fucα-1,3-)GlcNAc-β-1,3-Gal-β-1,4-(Fucα-1,3-)GlcNAc-β-1,3-Gal-β-1,4-(Fucα-1,3-)GlcNAc-β-1,2-Man-α-1,6-]Man-β-1,4-GlcNAc-β-1,4-GlcNAc-β-Asn	TE049
Gal-α-1,3-Gal-β-1,4-GlcNAc-β-1,3-Gal-β-1,4-GlcNAc-β-1,3-Gal-β-1,4-GlcNAc-β-1,3-Gal-β-1,4-GlcNAc-β-1,2-Man-α-1,3-(Gal-α-1,3-Gal-β-1,4-GlcNAc-β-1,3-Gal-β-1,4-GlcNAc-β-1,3-Gal-β-1,4-GlcNAc-β-1,3-Gal-β-1,4-GlcNAc-β-1,2-Man-α-1,6-)Man-β-1,4-GlcNAc-β-1,4-GlcNAc-β-Asn	TE050
GlcNAc-β-1,3-(GlcNAc-β-1,6-)Gal-β-1,4-Glc-Sp5	H0100
Gal-β-1,4-GlcNAc-β-1,3-(Gal-β-1,4-GlcNAc-β-1,6-)Gal-β-1,4-Glc-Sp5	H0101
Neu5Gc-α-2,6-Gal-β-1,4-GlcNAc-β-1,3-(Neu5Gc-α-2,6-Gal-β-1,4-GlcNAc-β-1,6-)Gal-β-1,4-Glc-Sp5	H0103
Fuc-α-1,2-Gal-β-1,4-GlcNAc-β-1,3-(Fuc-α-1,2-Gal-β-1,4-GlcNAc-β-1,6-)Gal-β-1,4-Glc-Sp5	H0105
Fuc-α-1,2-Gal-β-1,4-(Fuc-α-1,3-)GlcNAc-β-1,3-[Fuc-α-1,2-Gal-β-1,4-(Fuc-α-1,3-)GlcNAc-β-1,6-]Gal-β-1,4-Glc-Sp5	H0106
Gal-β-1,4-GlcNAc-β-1,3-(GlcNAc-β-1,6-)Gal-β-1,4-Glc-Sp5	H0200
Neu5Ac-α-2,6-Gal-β-1,4-GlcNAc-β-1,3-(GlcNAc-β-1,6-)Gal-β-1,4-Glc-Sp5	H0201
Neu5Gc-α-2,6-Gal-β-1,4-GlcNAc-β-1,3-(GlcNAc-β-1,6-)Gal-β-1,4-Glc-Sp5	H0202
Gal-β-1,4-(Fuc-α-1,3-)GlcNAc-β-1,3-(GlcNAc-β-1,6-)Gal-β-1,4-Glc-Sp5	H0203
Fuc-α-1,2-Gal-β-1,4-GlcNAc-β-1,3-(GlcNAc-β-1,6-)Gal-β-1,4-Glc-Sp5	H0204
Neu5Ac-α-2,6-Gal-β-1,4-GlcNAc-β-1,3-(Gal-β-1,4-GlcNAc-β-1,6-)Gal-β-1,4-Glc-Sp5	H0205
Gal-β-1,4-(Fuc-α-1,3-)GlcNAc-β-1,3-(Gal-β-1,4-GlcNAc-β-1,6-)Gal-β-1,4-Glc-Sp5	H0207
Fuc-α-1,2-Gal-β-1,4-GlcNAc-β-1,3-(Gal-β-1,4-GlcNAc-β-1,6-)Gal-β-1,4-Glc-Sp5	H0208
Fuc-α-1,2-Gal-β-1,4-(Fuc-α-1,3-)GlcNAc-β-1,3-(GlcNAc-β-1,6-)Gal-β-1,4-Glc-Sp5	H0209
Fuc-α-1,2-Gal-β-1,4-(Fuc-α-1,3-)GlcNAc-β-1,3-(GlcNAc-β-1,6-)Gal-β-1,4-Glc-Sp5	H0210

GlcNAc-β-1,3-(Gal-β-1,4-GlcNAc-β-1,6-)Gal-β-1,4-Glc-Sp5	H0300
GlcNAc-β-1,3-(Neu5Ac-α-2,6-Gal-β-1,4-GlcNAc-β-1,6-)Gal-β-1,4-Glc-Sp5	H0301
GlcNAc-β-1,3-[Gal-β-1,4-(Fuc-α-1,3-)GlcNAc-β-1,6-]Gal-β-1,4-Glc-Sp5	H0303
Fuc-α-1,2-GlcNAc-β-1,3-(Gal-β-1,4-GlcNAc-β-1,6-)Gal-β-1,4-Glc-Sp5	H0304
Gal-β-1,4-GlcNAc-β-1,3-(Neu5Ac-α-2,6-Gal-β-1,4-GlcNAc-β-1,6-)Gal-β-1,4-Glc-Sp5	H0305
Gal-β-1,4-GlcNAc-β-1,3-(Neu5Gc-α-2,6-Gal-β-1,4-GlcNAc-β-1,6-)Gal-β-1,4-Glc-Sp5	H0306
Gal-β-1,4-GlcNAc-β-1,3-[Gal-β-1,4-(Fuc-α-1,3-)GlcNAc-β-1,6-]Gal-β-1,4-Glc-Sp5	H0307
Gal-β-1,4-Glc-Sp	H0400
GalNAc-β-1,3-Gal-β-1,4-Glc-Sp	H0402
Neu5Ac-α-2,3-Gal-β-1,4-Glc-Sp	H0403
Neu5Gc-α-2,3-Gal-β-1,4-Glc-Sp	H0404
Neu5Ac-α-2,6-Gal-β-1,4-Glc-Sp	H0405
Neu5Gc-α-2,6-Gal-β-1,4-Glc-Sp	H0406
Gal-α-1,3-Gal-β-1,4-Glc-Sp	H0407
Neu5Ac-α-2,8-Neu5Ac-α-2,3-Gal-β-1,4-Glc-Sp	H0408
Neu5Ac-α-2,8-Neu5Ac-α-2,6-Gal-β-1,4-Glc-Sp	H0409
Neu5Ac-α-2,3-Gal-α-1,3-Gal-β-1,4-Glc-Sp	H0410
Neu5Ac-α-2,6-Gal-α-1,3-Gal-β-1,4-Glc-Sp	H0411
Gal-α-1,4-Gal-β-1,4-Glc-Sp5	H0500
GalNAc-β-1,3-Gal-α-1,4-Gal-β-1,4-Glc-Sp5	H0503
Gal-β-1,3-GalNAc-β-1,3-Gal-α-1,4-Gal-β-1,4-Glc-Sp5	H0504
Fuc-α-1,2-Gal-β-1,3-GalNAc-β-1,3-Gal-α-1,4-Gal-β-1,4-Glc-Sp5	H0505
Gal-β-1,4-GlcNAc-β-1,3-Gal-β-1,4-Glc-Sp5	H0600
Gal-β-1,4-(Fuc-α-1,3-)GlcNAc-β-1,3-Gal-β-1,4-Glc-Sp5	H0601
Fuc-α-1,2-Gal-β-1,4-GlcNAc-β-1,3-Gal-β-1,4-Glc-Sp5	H0602
GlcNAc-β-1,3-Gal-β-1,4-GlcNAc-β-1,3-Gal-β-1,4-Glc-Sp5	H0603
Neu5Ac-α-2,3-Gal-β-1,4-GlcNAc-β-1,3-Gal-β-1,4-Glc-Sp5	H0604
Gal-β-1,4-GlcNAc-β-1,3-(Neu5Ac-α-2,6-)Gal-β-1,4-Glc-Sp5	H0606
Fuc-α-1,2-Gal-β-1,4-(Fuc-α-1,3-)GlcNAc-β-1,3-Gal-β-1,4-Glc-Sp5	H0608

Neu5Ac-α-2,3-Gal-β-1,4-(Fuc-α-1,3-)GlcNAc-β-1,3-Gal-β-1,4-Glc-Sp5	H0609
GlcNAc-β-1,3-Gal-β-1,4-(Fuc-α-1,3-)GlcNAc-β-1,3-Gal-β-1,4-Glc-Sp5	H0610
Gal-β-1,4-GlcNAc-β-1,3-Gal-β-1,4-GlcNAc-β-1,3-Gal-β-1,4-Glc-Sp5	H0700
GlcNAc-β-1,3-Gal-β-1,4-GlcNAc-β-1,3-Gal-β-1,4-GlcNAc-β-1,3-Gal-β-1,4-Glc-Sp5	H0701
Gal-β-1,4-GlcNAc-β-1,3-Gal-β-1,4-GlcNAc-β-1,3-Gal-β-1,4-GlcNAc-β-1,3-Gal-β-1,4-Glc-Sp5	H0800
Gal-β-1,3-GlcNAc-β-1,3-(GlcNAc-β-1,6-)Gal-β-1,4-Glc-Sp5	H0900
Neu5Ac-α-2,3-Gal-β-1,4-Glc-Sp5	L1001
Neu5Gc-α-2,3-Gal-β-1,4-Glc-Sp5	L1002
Kdn-α-2,3-Gal-β-1,4-Glc-Sp5	L1003
Neu5Ac-a-2,3-(GalNAc-b-1,4-)Gal-b-1,4-Glc-Sp5	L1011
Neu5Gc-a-2,3-(GalNAc-b-1,4-)Gal-b-1,4-Glc-Sp5	L1012
Kdn-a-2,3-(GalNAc-b-1,4-)Gal-b-1,4-Glc-Sp5	L1013
Neu5Ac-a-2,3-(Gal-b-1,3-GalNAc-b-1,4-)Gal-b-1,4-Glc-Sp5	L1021
Neu5Gc-a-2,3-(Gal-b-1,3-GalNAc-b-1,4-)Gal-b-1,4-Glc-Sp5	L1022
Kdn-a-2,3-(Gal-b-1,3-GalNAc-b-1,4-)Gal-b-1,4-Glc-Sp5	L1023
Neu5Ac-α-2,8-Neu5Ac-α-2,3-Gal-β-1,4-Glc-Sp5	L1201
Neu5Gc-α-2,8-Neu5Ac-α-2,3-Gal-β-1,4-Glc-Sp5	L1202
Kdn-α-2,8-Neu5Ac-α-2,3-Gal-β-1,4-Glc-Sp5	L1203
Neu5Ac-α-2,8-Neu5Gc-α-2,3-Gal-β-1,4-Glc-Sp5	L1204
Neu5Gc-α-2,8-Neu5Gc-α-2,3-Gal-β-1,4-Glc-Sp5	L1205
Kdn-α-2,8-Neu5Gc-α-2-,3-Gal-β-1,4-Glc-Sp5	L1206
Neu5Ac-α-2,8-Kdn-α-2,3-Gal-β-1,4-Glc-Sp5	L1207
Kdn-α-2,8-Kdn-α-2,3-Gal-β-1,4-Glc-Sp5	L1209
Neu5Ac-α-2,8-Neu5Ac-α-2,3-(GalNAc-b-1,4-)Gal-β-1,4-Glc-Sp5	L1211
Neu5Gc-α-2,8-Neu5Ac-α-2,3-(GalNAc-b-1,4-)Gal-β-1,4-Glc-Sp5	L1212
Kdn-α-2,8-Neu5Ac-α-2,3-(GalNAc-b-1,4-)Gal-β-1,4-Glc-Sp5	L1213
Neu5Ac-α-2,8-Neu5Gc-α-2,3-(GalNAc-b-1,4-)Gal-β-1,4-Glc-Sp5	L1214
Neu5Gc-α-2,8-Neu5Gc-α-2,3-(GalNAc-b-1,4-)Gal-β-1,4-Glc-Sp5	L1215
Kdn-α-2,8-Neu5Gc-α-2,3-(GalNAc-b-1,4-)Gal-β-1,4-Glc-Sp5	L1216

Neu5Ac-α-2,8-Neu5Ac-α-2,3-(Gal-b-1,3-GalNAc-b-1,4-)Gal-β-1,4-Glc-Sp5	L1221
Neu5Gc-α-2,8-Neu5Ac-α-2,3-(Gal-b-1,3-GalNAc-b-1,4-)Gal-β-1,4-Glc-Sp5	L1222
Neu5Gc-α-2,8-Neu5Gc-α-2,3-(Gal-b-1,3-GalNAc-b-1,4-)Gal-β-1,4-Glc-Sp5	L1225
Kdn-α-2,8-Neu5Gc-α-2,3-(Gal-b-1,3-GalNAc-b-1,4-)Gal-β-1,4-Glc-Sp5	L1226
GlcNAc-β-1,3-Gal-β-1,4-Glc-Sp5	L2000
Gal-β-1,4-GlcNAc-β-1,3-Gal-β-1,4-Glc-Sp5	L2100
Gal-a-1,3-Gal-β-1,4-GlcNAc-β-1,3-Gal-β-1,4-Glc-Sp5	L2101
Gal-a-1,4-Gal-β-1,4-GlcNAc-β-1,3-Gal-β-1,4-Glc-Sp5	L2102
Neu5Ac-a-2,3-Gal-β-1,4-GlcNAc-β-1,3-Gal-β-1,4-Glc-Sp5	L2111
Neu5Gc-a-2,3-Gal-β-1,4-GlcNAc-β-1,3-Gal-β-1,4-Glc-Sp5	L2112
Kdn-a-2,3-Gal-β-1,4-GlcNAc-β-1,3-Gal-β-1,4-Glc-Sp5	L2113
Neu5Ac-a-2,8-Neu5Ac-a-2,3-Gal-β-1,4-GlcNAc-β-1,3-Gal-β-1,4-Glc-Sp5	L2121
Neu5Gc-a-2,8-Neu5Ac-a-2,3-Gal-β-1,4-GlcNAc-β-1,3-Gal-β-1,4-Glc-Sp5	L2122
Gal-β-1,4-(Fuc-α-1,3-)GlcNAc-β-1,3-Gal-β-1,4-Glc-Sp5	L2103
Gal-a-1,3-Gal-β-1,4-(Fuc-α-1,3-)GlcNAc-β-1,3-Gal-β-1,4-G	L2104
Neu5Ac-a-2,3-Gal-β-1,4-(Fuc-α-1,3-)GlcNAc-β-1,3-Gal-β-1,4-Glc-Sp5	L2131
Neu5Gc-a-2,3-Gal-β-1,4-(Fuc-α-1,3-)GlcNAc-β-1,3-Gal-β-1,4-Glc-Sp5	L2132
Kdn-a-2,3-Gal-β-1,4-(Fuc-α-1,3-)GlcNAc-β-1,3-Gal-β-1,4-Glc-Sp5	L2133
Fuc-α-1,2-Gal-β-1,4-GlcNAc-β-1,3-Gal-β-1,4-Glc-Sp5	L2191
GalNAc-α-1,3-(Fuc-α-1,2-)Gal-β-1,4-GlcNAc-β-1,3-Gal-β-1,4-Glc-Sp5	L2192
GlcNAc-β-1,3-Gal-β-1,4-GlcNAc-β-1,3-Gal-β-1,4-Glc-Sp5	L2200
Gal-β-1,4-GlcNAc-β-1,3-Gal-β-1,4-GlcNAc-β-1,3-Gal-β-1,4-Glc-Sp5	L2300
Gal-a-1,3-Gal-β-1,4-GlcNAc-β-1,3-Gal-β-1,4-GlcNAc-β-1,3-Gal-β-1,4-Glc-Sp5	L2301
Gal-a-1,4-Gal-β-1,4-GlcNAc-β-1,3-Gal-β-1,4-GlcNAc-β-1,3-Gal-β-1,4-Glc-Sp5	L2302
Neu5Ac-a-2,3-Gal-β-1,4-GlcNAc-β-1,3-Gal-β-1,4-GlcNAc-β-1,3-Gal-β-1,4-Glc-Sp5	L2311
Neu5Gc-a-2,3-Gal-β-1,4-GlcNAc-β-1,3-Gal-β-1,4-GlcNAc-β-1,3-Gal-β-1,4-Glc-Sp5	L2312
Gal-β-1,4-GlcNAc-β-1,3-Gal-β-1,4-(Fuc-α-1,3-)GlcNAc-β-1,3-Gal-β-1,4-Glc-Sp5	L2303
Gal-β-1,4-(Fuc-α-1,3-)GlcNAc-β-1,3-Gal-β-1,4-(Fuc-α-1,3-)GlcNAc-β-1,3-Gal-β-1,4-Glc-Sp5	L2304
Fuc-α-1,2-Gal-β-1,4-GlcNAc-β-1,3-Gal-β-1,4-GlcNAc-β-1,3-Gal-β-1,4-Glc-Sp5	L2391

GalNAc-α-1,3-(Fuc-α-1,2-)Gal-β-1,4-GlcNAc-β-1,3-Gal-β-1,4-GlcNAc-β-1,3-Gal-β-1,4-Glc-Sp5	L2392
Gal-β-1,3-GlcNAc-β-1,3-Gal-β-1,4-Glc-Sp5	L2900
Neu5Ac-a-2,3-Gal-β-1,3-GlcNAc-β-1,3-Gal-β-1,4-Glc-Sp5	L2911
Neu5Gc-a-2,3-Gal-β-1,3-GlcNAc-β-1,3-Gal-β-1,4-Glc-Sp5	L2912
Kdn-a-2,3-Gal-β-1,3-GlcNAc-β-1,3-Gal-β-1,4-Glc-Sp5	L2913
Gal-α-1,4-Gal-β-1,4-Glc-Sp5	L3100
GalNAc-β-1,3-Gal-α-1,4-Gal-β-1,4-Glc-Sp5	L3101
Gal-β-1,3-GalNAc-β-1,3-Gal-α-1,4-Gal-β-1,4-Glc-Sp5	L3102
Neu5Ac-a-2,3-Gal-β-1,3-GalNAc-β-1,3-Gal-α-1,4-Gal-β-1,4-Glc-Sp5	L3111
Neu5Gc-a-2,3-Gal-β-1,3-GalNAc-β-1,3-Gal-α-1,4-Gal-β-1,4-Glc-Sp5	L3112
Kdn-a-2,3-Gal-β-1,3-GalNAc-β-1,3-Gal-α-1,4-Gal-β-1,4-Glc-Sp5	L3113
Fuc-α-1,2-Gal-β-1,3-GalNAc-β-1,3-Gal-α-1,4-Gal-β-1,4-Glc-Sp5	L3103
Gal-α-1,3-Gal-β-1,4-Glc-Sp5	L3200
GalNAc-β-1,3-Gal-α-1,3-Gal-β-1,4-Glc-Sp5	L3201
Gal-β-1,3-GalNAc-β-1,3-Gal-α-1,3-Gal-β-1,4-Glc-Sp5	L3202
Neu5Ac-a-2,3-Gal-β-1,3-GalNAc-β-1,3-Gal-α-1,3-Gal-β-1,4-Glc-Sp5	L3211
Neu5Gc-a-2,3-Gal-β-1,3-GalNAc-β-1,3-Gal-α-1,3-Gal-β-1,4-Glc-Sp5	L3212
Kdn-a-2,3-Gal-β-1,3-GalNAc-β-1,3-Gal-α-1,3-Gal-β-1,4-Glc-Sp5	L3213

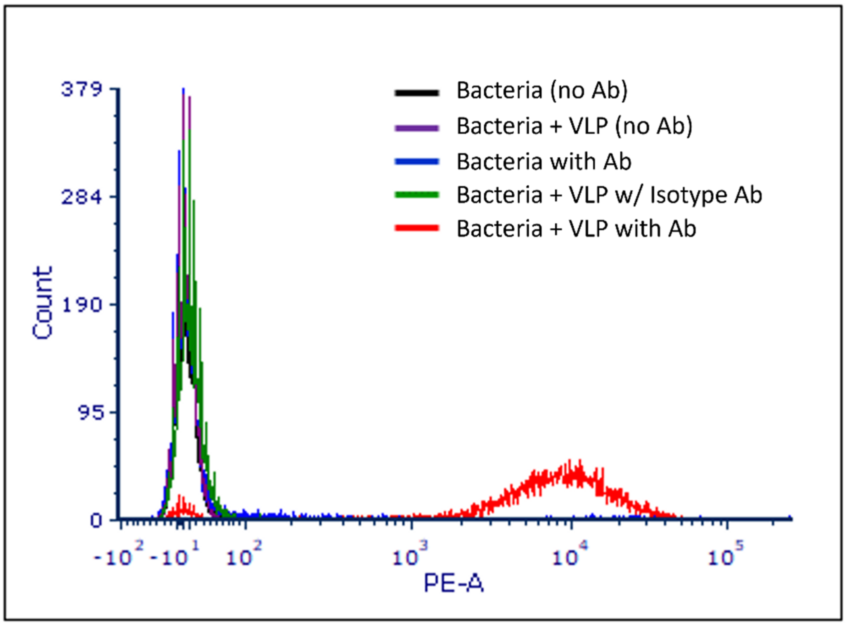

Bacteria (no Ab)
Bacteria + VLP (no Ab)
Bacteria with Ab
Bacteria + VLP w/ Isotype Ab
Bacteria + VLP with Ab
Count
379
284
190
95
0
-10^2 -10^1 10^2
10^3
10^4
10^5
PE-A

Supplemental Figure 1. Human Norovirus GII.4 VLP binding to *E. coli*. The histogram demonstrates detection of fluorescently labeled HuNoV antibody binding to HuNoV VLPs attached to commensal bacteria (red) with signal above the levels achieved for bacteria incubated without antibody (black), bacteria incubated with VLP and without antibody (purple), bacteria alone with antibody (blue), and bacteria with VLP treated with the antibody isotype control (green).
